# Variation of choroidal thickness and vessel diameter in patients with posterior non-infectious uveitis

**DOI:** 10.1186/s12348-014-0014-z

**Published:** 2014-08-16

**Authors:** Millena G Bittencourt, Saleema Kherani, Daniel A Ferraz, Mehreen Ansari, Humzah Nasir, Yasir J Sepah, Mostafa Hanout, Diana V Do, Quan Dong Nguyen

**Affiliations:** Retina Imaging Research and Reading Center, Wilmer Eye Institute, Johns Hopkins University, 1800 Orleans Street Woods 259-A, Baltimore, 21287 MD USA; Hospital das Clínicas, Universidade de São Paulo, Av. Dr. Enéas de Carvalho Aguiar, 255 Instituto Central, Cerqueira César, São Paulo, CEP 05403-900 SP Brazil; Ocular Imaging Research and Reading Center, Stanley M. Truhlsen Eye Institute, University of Nebraska Medical Center, 985540 Nebraska Medical Center, 3902 Leavenworth Street, Omaha, 68198-5540 NE USA

**Keywords:** Choroidal inflammatory disease, Choroidal thickness, Optical coherence tomography, Non-infectious uveitis and white dot syndrome

## Abstract

**Background:**

Choroidal thickness (CTh) and choroidal vessel diameter (VD) in the Haler’s layer were evaluated as markers of inflammatory insult in non-infectious uveitis (NIU). Spectral-domain optical coherence tomography (Spectralis®, Heidelberg Engineering Inc.) scans were acquired from 23 normal subjects (39 eyes – group 1), 7 subjects with high myopia (14 eyes – group 2), and 19 patients with NIU (23 eyes – group 3). In groups 1 and 2, CTh and VD were measured at 3 different points of the same horizontal OCT scan passing through the fovea and a mean calculated. Mean CTh and VD were calculated in 2 other locations, 2 mm superior and inferior from the chosen foveal horizontal scan. In group 3, three measurements of CTh and VD were obtained within 1 mm of a horizontal scan passing through a retinal lesion; mean CTh and VD were then computed. A ratio (*R*) between the VD and the corresponding CTh was calculated.

**Results:**

Group 1, 2 and 3 mean age was 29.6, 29.1 and 45.9 years, respectively. Sixteen normal subjects, three myopic subjects and six NIU patients were male.. Group 1 mean CTh did not differ from group 2 (261.6±45.6 vs. 260.2±50.6 µm µm; *p*>0.05); mean VD was marginally higher in Group 2 (159.8±32.2 vs. 163.2±33.2 µm; p>0.05). Group 3 demonstrated thinner CTh (193.6±54.6 µm) than Groups 1 and 2 (*p* = 0.02 and <0.001). Group 3 mean VD (123.6±37.4 µm) was also less than that in Groups 1 and 2; the difference was statistically significant only when compared to group 2, *p* = 0.01. R did not differ across groups (*p*-values >0.05), indicating that variations in CTh and VD followed the same trend.

**Conclusions:**

The study reports potential quantitative OCT-derived parameters that may be explored in future trials of non-infectious uveitis. Thinning of choroid and decrease of vessel diameter are observed in patients with chronic NIU compared to controls.

**Electronic supplementary material:**

The online version of this article (doi:10.1186/s12348-014-0014-z) contains supplementary material, which is available to authorized users.

## Background

Management of non-infectious uveitis (NIU) presents a unique challenge given the multitude of presentations, associated complications, and a lack of a definitive treatment thereof. Its pathophysiology and triggers are yet to be fully understood, consequently making it difficult to identify and observe biomarkers (anatomic and biochemical) that may help in early diagnosis and allow objective assessment of response to therapy.

Careful evaluation is required on part of the care providers to overcome the diagnostic challenge, which is often made by excluding a list of possible pathologies. Despite the great advances in diagnostic tests, a definitive diagnosis still remains elusive in more than 20% of uveitis cases [1]. In addition to infectious and autoimmune serologic assays, interdisciplinary evaluations, radiologic examination, ultrasonography, and fluorescein and indocyanine angiography, optical coherence tomography (OCT), a relatively recent imaging modality, has been used to accurately characterize structural damage to the retina and choroid in patients with NIU. It allows imaging of the retina and choroid with microscopic resolution, facilitating the differentiation and follow-up of subtle changes.

Various studies have been conducted to study the morphological changes seen in NIU employing OCT. Qualitative changes in the retinal and choroidal microstructures, such as choroidal, retinal pigment epithelium (RPE), and retinal intra-layer hyper reflectivity, have been described in many of the NIU diagnoses [2]-[4]. Despite the advances in the characterization of NIU provided by OCT, OCT-derived quantitative outcomes are yet to be evaluated as predictors of inflammation and functional outcomes.

Since inflammatory diseases of the choriocapillaris and stromal inflammatory vasculopathy play a role in the pathogenesis of many of the NIU conditions, changes in the normal blood circulation in the choroid are expected [5]-[9]. We hypothesized that inflammatory mediators produced in the course of these disorders may yield changes in choroidal thickness and vessel size and therefore could potentially be used as outcomes in future clinical trials. The purpose of our study was to quantify the role of vessel diameter and choroidal thickness as biomarkers of chronic inflammatory insults in non-infectious uveitis.

## Methods

Cross-sectional clinical and imaging data were retrospectively collected from patients in the Retina Division of the Wilmer Eye Institute, Johns Hopkins Hospital. Only spectral domain OCT images acquired with Spectralis HRA+OCT (version 1.5.12.0, Heidelberg Engineering Inc., Carlsbad, CA, USA) were used in the study. The standards of the Helsinki Declaration were followed; written informed consent was obtained from all participants after the study approval by the Johns Hopkins Hospital Research Ethics Committee has been granted.

Demographic information was collected. Spectral domain optical coherence tomography (SD-OCT) images from participants were divided into three groups: group 1 (normal), group 2 (high myopia), and group 3 (NIU). Images from normal volunteers with no known ocular diseases and spherical equivalent refractive error >−6.00 D were assigned to group 1. As some patients in the NIU cohort had multifocal choroiditis (MFC) and punctate inner choroidopathy (PIC), which are commonly associated with high myopia, a cohort with highly myopic individuals was included in the study. Highly myopic individuals should have spherical equivalent refractive error ≤−6.00 D and fundus changes compatible with myopia and no other pathology associated. Subjects assigned to group 3 had their diagnoses confirmed after extensive evaluation by a retina and uveitis specialist (QDN). Among the ancillary studies employed to reach the diagnosis were ophthalmic examinations, infectious and autoimmune serologic assays, radiologic images, and interdisciplinary consultation when required, as well as SD-OCT and fluorescein angiography. Subjects <18 years old and with any other ocular pathology different from chronic NIU were excluded from the study.

**Figure 1:**
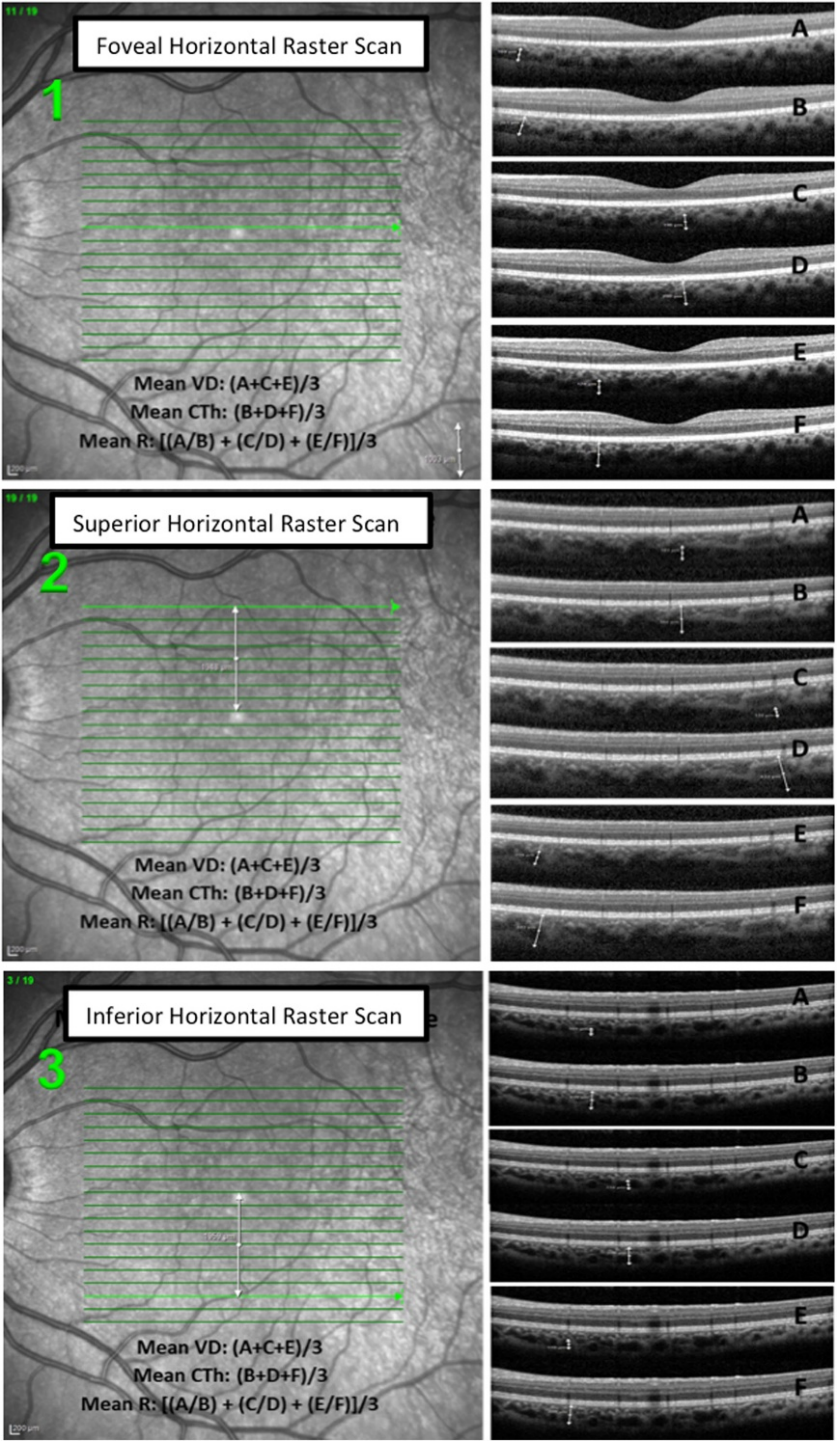
**Measurement algorithm.** Measurement algorithm in the OCT of a normal subject. A: VD measurement 1, B: CTh measurement 1, C: VD measurement 2, D: CTh measurement 2, E: VD measurement 3, F: CTh measurement 3

OCT images with signal-to-noise ratio >15 were included in the study. Images used for analysis followed a standardized OCT scanning protocol. All images were acquired using 20° × 15° rectangle with horizontal raster scans, each comprised at least ≥16 frames per b-scan, encompassing the macula and optic nerve temporal border. Manual calipers were used to measure choroidal thickness (CTh) from the outer hyper-reflective border of the base of the retinal pigment epithelium (RPE) - RPE/BM complex - to the hypo-reflective inner border of the sclera (Figure 1) [10]. The choroidal layer was further subdivided into Haller's large vessel layer (HLVL) and Sattler's medium vessel layer (SMVL). Choroidal vessel diameter (VD) was measured in the HLVL of the choroid, which is defined as outer choroid large hypo-intense spaces, representing large vascular luminal spaces [8]. The imaging measurement algorithm is illustrated in Figure 1.

In normal and myopic subjects, CTh and VD were measured at three different points of the same OCT horizontal line and a mean was calculated. Each subject had the mean CTh and VD calculated for three horizontal lines: one sub-foveal, one 2 mm superior, and one 2 mm inferior from the chosen foveal horizontal scan. These three locations were used to compute a mean macular CTh and VD and served as a reference to analyze the CTh and VD of group 3.

In subjects with NIU, retinochoroidal lesions within any of the macular quadrants were identified, and the mean CTh and VD were obtained from three measurements of a given horizontal line passing through each lesion.

A ratio (*R*) between the VD measurement and the corresponding CTh measurement was calculated (*R* = VD/CTh) and used for comparison among normal, myopic, and NIU patients.

### Statistical analysis

The SPSS (IBM® Inc., Chicago, IL, USA) release 19.0.0 was used for statistical analysis. Demographic characteristics of the patients were summarized using descriptive statistics and expressed as mean and 95% confidence intervals. Exploratory data analysis (EDA) was done to verify the data set characteristics, including covariates, and to orient the comparison analysis.

A multilevel analysis using a general model with random intercepts, which took into consideration age, spherical equivalent refractive error, measurement location, gender, and ethnicity, was used to compare the CTh, VD, and ratio among the three groups. In addition, as the mean CTh, VD, and *R* for each horizontal line for the same individual were analyzed individually, the model was adjusted to assume that there would be a correlation of retinal scans at two different levels: eyes and patients. Therefore, a random intercept for each of these two levels was also included. Kruskal-Wallis test was used to evaluate potential differences across different diagnoses of NIU. Similarly, Mann-Whitney test was conducted to compare the CTh, VD, and ratio between pairs of diagnosis. Mean and confidence interval were calculated to better visualize the possible overlapping distributions. A *p* value of <0.05 was considered statistically significant.

## Results

Among the subjects enrolled, 23 had no known ocular pathology (39 eyes), 7 were highly myopic without any other retinal pathology (14 eyes), and 19 were patients with various types of NIU (23 eyes). Sixteen (51.6%) of the normal subjects, three (50%) of the myopic subjects, and six (35.2%) of the NUI patients were male. Mean age for normal, myopic, and NIU patients was 29.6, 29.1, and 45.9 years, respectively. The mean spherical refractive error in groups 1, 2, and 3 was −1.80 D (SD ±1.4), −7.30 D (SD ± 2.6), and −4.68 D (SD ±4), respectively. The best corrected visual acuity in ETDRS letters was 84 ± 3.6 in group 1, 75 ± 6.4 in group 2, and 69 ± 17 in group 3. In group 3, eight eyes were from patients diagnosed with multifocal choroiditis (MFC), four from patients with punctate inner choroidopathy (PIC), six from patients with birdshot chorioretinopathy (BSCR), three from patients with acute zonal occult outer retinopathy (AZOOR), while one eye was from a patient with serpiginous chorioretinitis (SC) and one with Vogt-Koyanagi-Harada (VKH) syndrome.

The NIU mean duration was 7 (±4.6 SD) years. All patients were in disease remission by the time of the study visit (for the analyses in this study). Four eyes (17.4%) were in remission for at least 2 years, ten eyes (43.5%) were in remission for at least 1 year, six eyes (26.1%) were in remission for at least 6 months, and three eyes (13%) were in remission for more than 1 month but less than 6 months. The mean time of controlled disease was 15.3 (±12.3 SD) months, 60.9% of the eyes were in remission for at least 1 year and 39.1% were in remission for less than 1 year. Twelve patients were on immunomodulatory therapy (IMT) alone, three patients were on both IMT and corticosteroids, three patients were taking less than 10 mg/day of prednisone as monotherapy, and one was not on any treatment. The 23 eyes were considered as having chronic disease in remission at the time of the study. Three patients had bilateral and 16 patients had unilateral ocular involvement. Demographic data and clinical characteristics of study subjects are summarized in Table 1.

**Table 1:** **Characteristics of study subjects**

	***N***eyes	Age		Gender		Ethnicity			Refractive error	
		Years	SD	Male	Female	C	AF	A	Spherical equivalent	SD
Group 1	39	29.6	7.4	18	18	18	4	17	−1.8	1.40
Group 2	14	29.1	8.7	8	6	4	2	8	−7.3	2.60
Group 3	23	45.9	14.4	7	16	19	3	1	−4.68	4.00
MFC	8	44.4	8.5	2	6	5	2	1	−5.54	3.50
PIC	4	33.6	3.3	2	2	4	0	0	−8.86	2.30
AZOOR	3	56.0	20.7	0	3	3	0	0	−1.16	0.28
BSCR	6	63.8	9.0	1	5	6	0	0	−1.30	3.00
VKH	1	25	0	1	0	0	1	0	−3.00	0.00
SC	1	38	0	1	0	1	0	0	−1.00	0.00

Group 1: normal subjects; group 2: highly myopic subjects; group 3: subjects with non-infectious uveitis, multifocal choroiditis (MFC), punctate inner choroidopathy (PIC), acute zonal occult outer retinopathy (AZOOR), birdshot choroidoretinopathy (BSCR), Vogt-Koyanagi-Harada syndrome (VKH), serpiginous choroiditis (SC). C, Caucasian; AF, African; A, Asian.

The mean CTh was similar in normal subjects, 261.6 ± 45.6 μm, and myopic subjects, 260.2 ± 50.6 μm. Patients with NIU had thinner mean CTh, 193.6 ± 54.6 μm, as compared to the control groups. The mean VD was found to be larger in myopic subjects, 163.2 ± 33.2 μm, than in normal subjects and patients with NIU, 159.8 ± 32.2 μm and 123.6 ± 37.4 μm, respectively. Groups 1, 2, and 3 had approximately the same mean ratio, 0.61 ± 0.1, 0.61 ± 0.05, and 0.62 ± 0.01, respectively (*p* > 0.05) (Table 2).

**Table 2:** **Choroidal thickness, vessel diameter, and ratio characteristics**

	Choroidal thickness (μm)				Vessel vertical diameter (μm)				Ratio			
	Mean	SE	95% CI		Mean	SE	95% CI		Mean	SE	95% CI	
			Lower	Upper			Lower	Upper			Lower	Upper
Group 1	261.6	4.5	253.1	271.3	159.8	3.2	154.0	166.2	0.61	0.01	0.59	0.65
Group 2	260.2	7.8	244.5	275.5	163.2	5.1	152.9	174.1	0.62	0.00	0.61	0.64
Group 3	193.6	8.5	176.9	213.3	123.6	5.8	111.7	135.5	0.61	0.01	0.60	0.67
MFC	187.7	10.4	165.3	210.0	123.1	8.8	104.2	142.0	0.65	0.02	0.6	0.69
PIC	198.4	16.9	159.3	237.5	127.6	11.5	101.0	154.3	0.64	0.02	0.58	0.70
AZOOR	318.8	18.2	240.2	397.3	177.3	13.5	119.1	235.5	0.55	0.04	0.36	0.74
BSCR	160.4	7.1	143.8	177.1	98.2	8.9	77.5	118.9	0.61	0.04	0.51	0.71
VKH	192.5	3.5	148.0	236.9	130.5	26.5	104.0	157.0	0.68	0.15	0.53	0.83
SC	183.2	66.2	117.0	249.5	137.5	45.5	92.0	183.1	0.75	0.02	0.73	0.79

Group 1: normal subjects; group 2: highly myopic subjects; group 3: subjects with non-infectious uveitis, multifocal choroiditis (MFC), punctate inner choroidopathy (PIC), acute zonal occult outer retinopathy (AZOOR), birdshot choroidoretinopathy (BSCR), Vogt-Koyanagi-Harada syndrome (VKH), serpiginous choroiditis (SC).

In normal subjects, the mean CTh has shown to be greater towards the arcades (mean foveal CTh = 242.8 ± 37.5 μm, mean supra-foveal CTh = 271.8 ± 50.4 μm, mean infra-foveal CTh = 275.3 ± 43 μm; *p* < 0.05). Mean VD was larger superiorly (173.9 ± 36.8 μm) than inferiorly (159.5 ± 26.6 μm, *p* > 0.06) and in the fovea (148.5 ± 28.1 μm, *p* < 0.001); the difference between mean VD in the fovea and inferiorly was not significant (*p* = 0.16). The variation of *R* across subfield was not statistically significant, *p* values >0.05. The CTh, VD, and *R* values have not shown statistically significant differences between macular locations in group 2, which may indicate a more homogeneous thickness across myopic retinal subfields (Tables 3 and 4).

**Table 3:** **Choroidal thickness, vessel diameter, and ratio characteristics according to location in the fovea**

	Locations	Normal subjects			Highly myopic subjects		
		***N***	Mean	SD	***N***	Mean	SD
Mean CTh	Foveal	39	242.8	37.5	14	248.1	42.0
	Supra-foveal	32	271.8	50.4	14	273.1	58.6
	Infra-foveal	30	275.3	43.0	14	259.6	50.7
Mean VD	Foveal	39	148.5	28.1	14	152.6	26.9
	Supra-foveal	32	173.9	36.8	14	174.8	37.0
	Infra-foveal	30	159.5	26.6	14	162.4	33.5
Mean ratio	Foveal	39	0.614	0.086	14	0.618	0.055
	Supra-foveal	32	0.660	0.242	14	0.644	0.059
	Infra-foveal	30	0.582	0.150	14	0.628	0.065

Supra-foveal is defined as 2 mm superiorly to the fovea; infra-foveal is defined as 2 mm inferiorly to the fovea.

**Table 4:** **Choroidal thickness, vessel diameter, and ratio characteristics according to comparison of locations in the fovea**

	Comparison of locations	Normal subjects				Highly myopic subjects			
		Mean difference	95% CI		***p***value	Mean difference	95% CI		***p***value
			Lower bound	Upper bound			Lower bound	Upper bound	
Mean CTh	Foveal × supra-foveal	−27.9	−47.5	−8.4	0.006	−25.0	−64.4	14.4	0.206
	Foveal × infra-foveal	−30.6	−50.6	−10.6	0.003	−11.5	−50.9	27.9	0.557
	Supra-7foveal × infra-foveal	−2.7	−23.5	18.2	0.800	13.5	−25.9	52.9	0.492
Mean VD	Foveal × supra-foveal	−25.1	−39.6	−10.5	0.001	−22.2	−47.6	3.2	0.085
	Foveal × infra-foveal	−10.5	−25.3	4.3	0.162	−9.8	−35.1	15.6	0.440
	Supra-foveal × infra-foveal	14.6	−0.9	30.1	0.065	12.4	−13.0	37.8	0.328
Mean ratio	Foveal × supra-foveal	−0.048	−0.119	0.022	0.176	0.026	−0.026	−0.026	0.253
	Foveal × infra-foveal	0.028	−0.044	0.100	0.446	0.016	−0.010	−0.010	0.482
	Supra-foveal × infra-foveal	0.076	0.001	0.151	0.057	−0.016	−0.062	0.030	0.482

Supra-foveal is defined as 2 mm superiorly to the fovea; infra-foveal is defined as 2 mm inferiorly to the fovea.

The relationship between age, refractive error, gender, ethnicity, and the study variables were analyzed using multiple regression analysis. An increase of 1 year has shown to decrease the CTh by 1.7 μm and the VD by 0.98 μm, *p* values <0.001. Conversely, the decrease of 1 diopter has shown to increase the CTh by 3.6 μm (Table 5). Ethnicity was found to have no association with CTh, VD, and *R*. In group 3, the association between remission duration and CTh did not show significant strength (*r*^2^ = 0.389, *p* value = 0.96); therefore, this variable was not included in the model. Likewise, the association between remission duration and vessel diameter was also not significant (*r*^2^ = 0.276, *p* value = 1.02).

**Table 5:** **Association of different co-factors with choroidal thickness and vessel diameter**

	Choroidal thickness (μm)		Vessel vertical diameter (μm)	
	Effect	***p***value	Effect	***p***value
Age (⬆ 1 year)	−1.7	*<0.001*	−0.98	*<0.001*
Refractive error (⬇ 1 D)	3.6	*0.01*	0.85	0.390
Gender (M)	18.9	*0.07*	8.18	0.100
Ethnicity	-	0.22	-	0.666

D, diopter, M, male.

The comparisons among groups were adjusted for possible differences in age, refractive error, and gender, and for intra-eye and intra-patient correlation of repeated measurements. Mean CTh was slightly thicker in group 1 as compared to group 2; the difference, however, did not differ significantly (*p* value >0.05). Conversely, VD was higher in group 2, but not statistically significant (*p* = 0.92). Group 3 demonstrated thinner choroid when compared to groups 1 and 2. The difference was statistically significant with *p* values <0.05. The VD was also smaller in this group compared to groups 1 and 2; however, the difference was statistically significant only when compared to group 2, *p* = 0.008. The thin CTh of group 3 was not reflected by its group ratio when compared to groups 1 and 2, *p* value >0.05 (Table 6).

**Table 6:** **Group comparisons**

	Choroidal thickness (μm)		Vessel vertical diameter (μm)		Ratio	
	Difference	***p***value	Difference	***p***value	Difference	***p***value
Group 1 vs. group 2^a^	18.84	0.29	−8.1	0.92	0.029	1.00
Group 1 vs. group 3^a^	31.3	*0.02*	17.96	0.07	0.006	1.00
Group 2 vs. group 3^a^	50.2	*<0.001*	26.0	*0.01*	−0.023	0.31

^a^Multivariate model using age and refractive error as covariates (age = 33.16 and Rx = −3.7). Bonferroni test was used to adjust for multiple comparisons.

**Figure 2:**
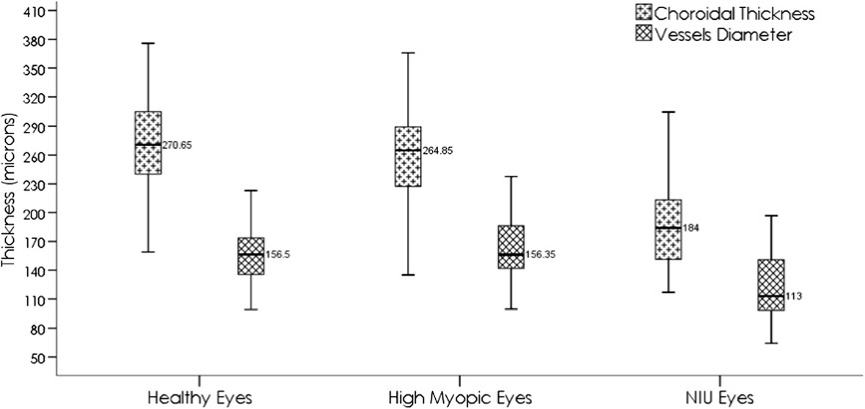
**Group choroidal thickness and vessel diameter intervals.** Choroidal thickness and vessel diameter intervals showing the difference between group 1 and group3 and between group 2 and group 3.

**Figure 3:**
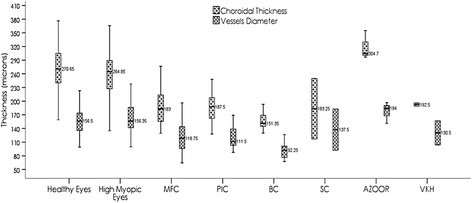
**Non-infectious uveitis choroidal thickness and vessel diameter intervals.** Choroidal thickness and vessel diameter intervals for different entities of non-infectious uveitis (NIU), showing the differences among AZOOR and other diagnosis.

**Figure 4:**
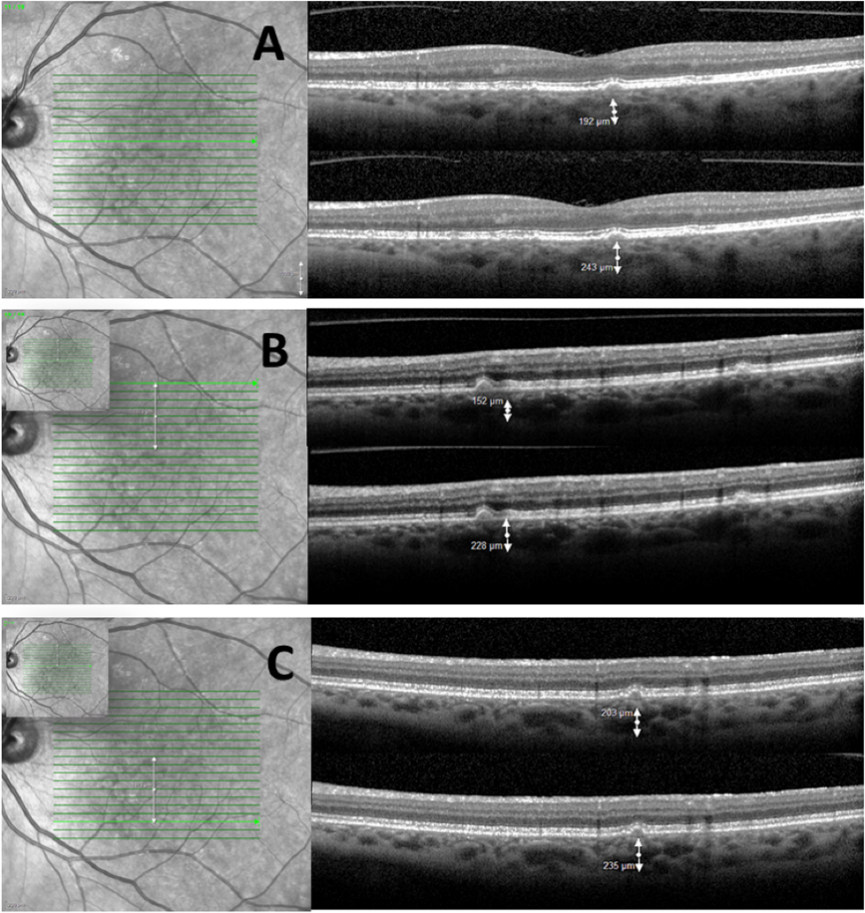
**Patient with birdshot retinochoroidopathy.** Spectral domain OCT of the left eye of a patient with birdshot retinochoroidopathy. The infrared SLO images **(A to C)** show the location of the horizontal b-scan. The correspondent b-scans show the vessel diameter and the choroidal thickness below the different lesions.

Among patients with NIU, the mean CTh was found to be greatest in the AZOOR patients (318.8 μm) followed by patients with PIC (198.4 μm), VKH (192.5 μm), MFC (187.7 μm), SC (183.2 μm), and finally BSCR (160.4 μm). The CTh difference among the various entities was significant (*p* = 0.04). Mean VD was largest in AZOOR patients (177.3 μm), followed by SC (137.5 μm), VKH (130.5 μm), PIC (127.6 μm), MFC (123.1 μm), and BSCR (98.2 μm); however, the differences among subgroup were not significant (*p* = 0.06). *R* was higher in patients with SC (0.75) followed by VKH (0.68), MFC (0.65), PIC (0.64), BSCR (0.61), and then AZOOR (0.55), *p* = 0.23. The complete description of the CTh, VD, and ratio distribution across patients with different NIU diagnoses is assembled in Table 2. The CTh and VD intervals are illustrated in Figures 2 and 3. Figure 4 shows the measurements of the VD and CTh of a patient with BSCR.

Mean differences in CTh and VD were found to be significant only across AZOOR patients and patients with MFC, PIC, and BSCR, *p* values <0.05. The *R* did not reflect any differences among subgroups (Table 7).

**Table 7:** **Comparisons between NIU diagnoses**

	Choroidal thickness (μm)		Vessel vertical diameter (μm)		Ratio	
Among NIU^a^	***p***value = 0.04		***p***value = 0.06		***p***value = 0.23	
Pair-wise comparison^b^	***Z***	***p***value	***Z***	***p***value	***Z***	***p***value
MFC vs. PIC	−0.48	0.63	−0.28	0.80	−0.05	0.97
MFC vs. AZOOR	−2.66	0.01	−2.23	0.02	−1.67	0.10
MFC vs. BSCR	−1.41	0.16	−1.72	0.08	−0.45	0.67
PIC vs. AZOOR	−2.49	0.01	−1.75	0.07	−1.57	0.11
PIC vs. BSCR	−1.76	0.07	−2.16	0.03	−0.30	0.75
AZOOR vs. BSCR	−2.50	0.01	−2.50	0.01	−1.20	0.22

^a^Kruskal-Wallis test; ^b^Mann-Whitney test. VKH and SC were not used for comparison due to the small number of subjects enrolled (1).

## Discussion

In this cross-sectional study, OCT-derived parameters were employed to quantitatively evaluate choroidal changes in a variety of posterior NIUs. The NIU cohort has shown demographic features in accordance with those described in the literature for patients with different white dot syndromes (WDSs). The strong female predilection seen in MFC and PIC, as well as the high incidence in the third and fourth decades, was confirmed in the study cohort [11]. Similarly, patients with BSCR, SC, AZOOR, and VKH have revealed characteristics similar to those previously described for these pathologies [12],[13].

Variation of CTh and VD were found across different subfields of the macula. This difference may be explained by physiological and anatomical differences between the retina fields. Light-dark adaptation, exercise, and blue-flickering stimulus have been shown to induce choroidal blood flow responses and diversely influence the CTh and VD across various locations in the posterior pole [14]. It is not unusual to find the thickest portion of the choroid to be underneath the fovea, where the oxygen requirement is highest, thus requiring high blood flow [15]. In addition, peripheral, equatorial, and sub-macular choroid has been shown to differ on the density of capillaries and inter-capillary space, being more compacted and thicker in the macula.

Recent studies of macular CTh employing enhanced depth imaging (EDI)-OCT have made known that sub-foveal CTh decreases rapidly in the nasal direction and less impressively in the temporal direction [10],[16]. In our study, the mean foveal CTh was averaged from three measurements taken at any point of the foveal horizontal scan, including the nasal and temporal subfields. Therefore, the overall foveal CTh found in our cohort was thinner than the central foveal CTh reported by other authors [10],[16],[17]. However, by using such method, the averaged foveal CTh could then be used as reference to evaluate uveitis lesions occurring at any point of the foveal horizontal scan.

The mean CTh measured in normal OCTs was found to be within the normal range described by Ding et al. In this study of 420 eyes (mean CTh = 261.93 ± 88.42 μm), the thickness range was found to be very close to the one reported by Fujiwara and colleagues in a study with 145 eyes (mean CTh 265.5 ± 82.4 μm) [18],[19].

The cohort of patients with NIU showed thinner CTh and VD than the normal and myopic controls. These findings support the hypothesis that inflammatory and ischemic insults to the choroid may occur during posterior non-infectious uveitis or may lead to atrophic changes in the choroidal layers [5],[6],[20],[7]. The discrepancy in the CTh and VD measurements between groups 2 and 3 shows that co-existing myopia in NIU patient is not a confounding factor in final NIU thickness.

Not surprisingly, some variations on the intensity of CTh and VD atrophy occur across different diagnosis of NIU. Most likely, it reflects the distinct inflammatory profile involved in pathophysiology of each entity as well as the variable vascular involvement.

Recently, Aoyagi and colleagues have reported two cases of Japanese patients with multiple evanescent white dot syndrome (MEWDS) who had CTh decrease from the acute to convalescent phase [21]. Similarly, Karampelas and collaborators have described loss of hyporeflectivity in HLVL and thinning of both choroid and HLVL layer in patients with panuveitis [8]. Furthermore, Keane and colleagues evaluated qualitative and quantitative choroidal changes in patients with BSCR in comparison to normal controls using enhanced depth OCT scans. Their study revealed generalized choroidal thinning, absence of Sattler's layer, discrete hyper-reflective foci, focal depigmentation, and the presence of supra-choroidal hypo-reflective space [4]. The present study supports these previous findings; the choroid of patients with BSCR, SC, and MFC have shown more profound thinning than those with PIC and VKH, when compared to normal and highly myopic controls. Even though it is still controversial whether AZOOR should be among the so-called white dot syndromes, it is well known that the majority of the pathologic insults to the fundus structures occur at the level of RPE and photoreceptor layers, more commonly sparing the choroidal layers [22],[23]. Normal CTh and VD are expected findings for AZOOR, yet it was not seen in our cohort. Our patients with AZOOR have shown thicker CTh and VD than normal and highly myopic controls. There are two conceivable explanations: the small number of subjects with AZOOR, which may not be representative of the diseases as a whole, and subclinical acute inflammation leading to the thickening effect described by Aoyagi and colleagues. Either explanations warrant further evaluation.

The ratio of VD and CTh did not vary significantly across groups or even within different NIU diagnoses. Changes in VD followed the overall choroidal thickness changes. In addition, our cohort was characterized by patients under effective immunomodulation and therefore was composed of patients with inactive disease. As described by Aoyagi, vessel dilatation or increased CTh is more expected in acute inflammatory phase, which was not the case in the major part of patients enrolled in our study. The present study shows that the ratio could not accurately characterize NIU damage.

To our knowledge, our study is the first to evaluate CTh and VD in various forms of NIU and compared to controls such as myopic patients and normal subjects. CTh and VD thinning have shown to be altered in the remission phase of various NIU and should be further explored as a potential parameter to monitor disease regression or progression. Our next step is to validate a semi-automated method to measure CTh and vessel volume.

There are several study limitations, including the small number of patients in the subgroup of NIU and the use of standard SD-OCT images. As the study collected data retrospectively, the availability of OCTs using EDI was limited; therefore, it was not incorporated in the study design. However, this limitation was considered an intrinsic factor, common to all images, which does not affect the validity of the results [15],[10],[24].

## Conclusions

The present study reports potential OCT-derived parameters in patients with non-infectious uveitis. We detected thinning of choroid and decrease of vessel diameter when compared to matched controls, which indicates that choroidal thickness and vessel size may serve as potential (follow-up) parameters to monitor patients with non-infectious uveitis in future studies. Further analysis with a larger sample size and semi-automated measurements is indicated to evaluate the role of choroidal thickness and vessel diameter in the management of active and inactive uveitic diseases.

## Authors’ original submitted files for images

Below are the links to the authors’ original submitted files for images. Authors’ original file for figure 1 Authors’ original file for figure 2 Authors’ original file for figure 3 Authors’ original file for figure 4

## Abbreviations

AZOOR: acute zonal occult outer retinopathy
BSCR: birdshot choroidoretinopathy
CTh: choroidal thickness
EDI: enhanced depth imaging
FA: fluorescein angiography
HLVL: Haller's large vessel layer
MFC: multifocal choroiditis
OCT: optical coherence tomography
PIC: punctate inner choroidopathy
RPE: retinal pigment epithelium
SC: serpiginous choroiditis
SMVL: Sattler's medium vessel layer
VD: vessel diameter
VKH: Vogt-Koyanagi-Harada syndrome
WDS: white dot syndrome
