# Supplementary figures and images for: Variation of choroidal thickness and vessel diameter in patients with posterior non-infectious uveitis
Source: J Ophthalmic Inflamm Infect. 2014 Aug 16;4:14. doi: 10.1186/s12348-014-0014-z (PMC4884007; doi:10.1186/s12348-014-0014-z)

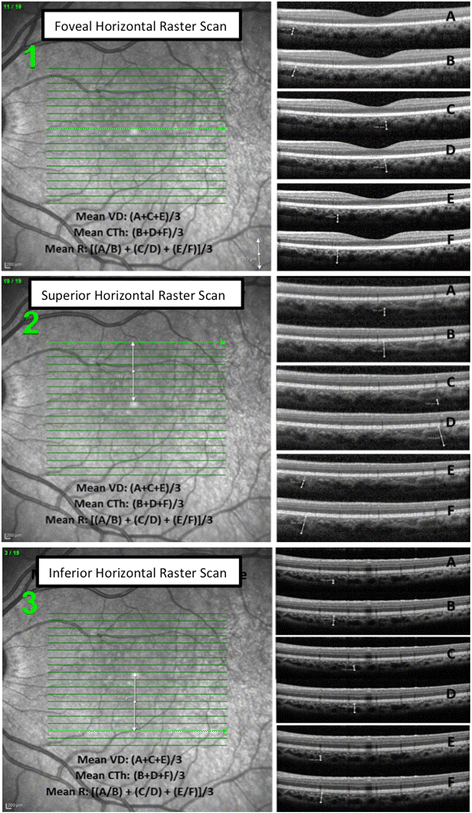

Supplement: Supplementary file 1 — Authors’ original file for figure 1 [file 12348_2014_14_MOESM1_ESM.gif]

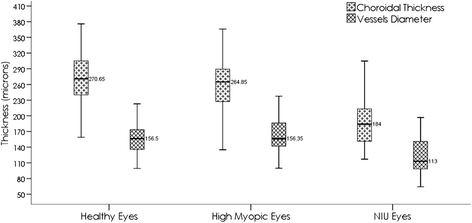

Supplement: Supplementary file 2 — Authors’ original file for figure 2 [file 12348_2014_14_MOESM2_ESM.gif]

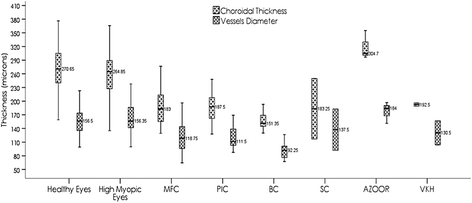

Supplement: Supplementary file 3 — Authors’ original file for figure 3 [file 12348_2014_14_MOESM3_ESM.gif]

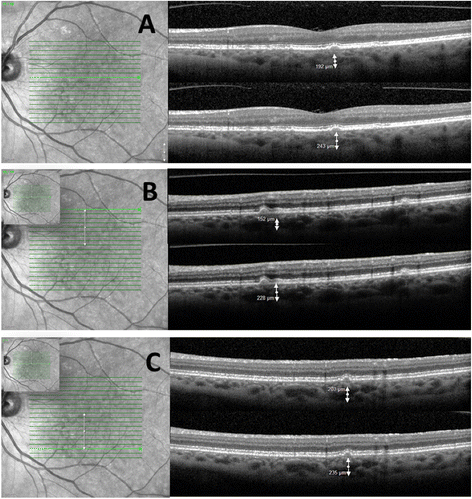

Supplement: Supplementary file 4 — Authors’ original file for figure 4 [file 12348_2014_14_MOESM4_ESM.gif]
